# Effects of Sevoflurane on Young Male Adult C57BL/6 Mice Spatial Cognition

**DOI:** 10.1371/journal.pone.0134217

**Published:** 2015-08-18

**Authors:** Jianhui Liu, Xiaoqing Zhang, Wei Zhang, Guojun Gu, Peijun Wang

**Affiliations:** 1 Department of Anesthesiology, Tongji Hospital affiliated Tongji University, Shanghai, China; 2 Department of medical image, Tongji Hospital affiliated Tongji University, Shanghai, China; Université Pierre et Marie Curie, FRANCE

## Abstract

Inhalation anesthetics are reported to affect cognition in both animals and humans. The influence of inhalation anesthetics in learning and memory are contradictory. We therefore investigated the effects of sevoflurane anesthesia with different durations on cognitive performance and the levels of NMDA receptor subunit NR2B, phosphorylated ERK1/2 (p-ERK1/2) and activated caspase3 in mouse hippocampus. We anaesthetized eight-week old male C57BL/6 mice with 2.5% sevoflurane for durations ranging from one to four hours. Non-anaesthetized mice served as controls. Mice exposed to sevoflurane for one to three hours showed improved performance, whereas mice with exposure up to four hours displayed similar behavioral performance as control group. NR2B was increased both at 24h and at two weeks post sevoflurane exposure in all groups. The p-ERK1/2: total ERK1/2 ratio increased at 24h in all anesthesia groups. The ratio remained elevated at two weeks in groups with two- to four-hour exposure. Activated caspase3 was detected elevated at 24h in groups with two- to four-hour exposure. The elevated trend of activated caspase3 was still detectable at two weeks in groups with three- to four-hour exposure. At two weeks post anesthesia, the typical morphology associated with apoptotic cells was observed in the hippocampus of mice exposed to four hours of sevoflurane. Our results indicate that 2.5% sevoflurane exposure for one to three hours improved spatial cognitive performance in young adult mice. The cognitive improvement might be related to the increase of NR2B, the p-ERK1/2: total ERK1/2 ratio in hippocampus. However, exposure to sevoflurane for four hours caused neurotoxicity due to caspase3 activation and apoptosis.

## Introduction

Animal and human studies proved that inhalation anesthetics affect cognitive function[1–6]. Their short term effects as amnesic agents are well known, while their long term effects, and especially their pro-cognitive effects need to be better presented. Some investigators reported that isoflurane-induced cognitive impairments in rats[1–3], whereas others described cognitive improvements following isoflurane exposure in rodents up to 2 weeks after isoflurane anesthesia[1,4,5]. Sevoflurane, with low blood/gas ratio, fast onset and recovery and low pungency[7–9] is more widely used for induction and maintenance of general anesthesia during surgery than other inhalation anesthetics. While there is still a lack of studies concerning sevoflurane’s effect on cognition. Several studies reported sevoflurane neurotoxicity and cognitive impairment focused on neonatal mice [10–12]. While the effects of sevoflurane on adult mice cognition are fewer reported. It is recently reported that sevoflurane anesthesia induced even an improvement of adult mice cognitive performance 24 hours after anesthesia[13]. But we still have no information about how long these effects last and how they have been interpreted.

Although the exact mechanism of commonly used inhalation anesthetic sevoflurane remains unclear, increasing evidence supports the theory that inhalation anesthetics act on postsynaptic receptors. Many inhalational anesthetics, such as halothane, isoflurane and sevoflurane potentiate inhibitory ion channels (i.e., GABA_A_ receptors) and inhibit excitatory ion channels (i.e., NMDA receptors), which contribute to the reduction of neuronal excitability and lead to anesthesia [14,15].

Extracellular signal-regulated kinase 1 and 2 (ERK1/2) are the key players downstream of NMDA receptor activation. Upregulation of phosphorylated ERK1/2 (p-ERK1/2) upon NMDA stimulation promotes neuronal survival, whereas downregulation of p-ERK1/2 leads to severe neurodegeneration[16,17]. Sevoflurane has been reported to inhibit the phosphorylation of extracellular signal-regulated kinase 1 and 2 (ERK1/2) in the hippocampal tissues [18]. Due to the critical role of NMDA receptors and its downstream signaling molecules in learning and memory processes[19], these receptors may play a role in sevoflurane-induced cognition change in brain. Therefore, in this study, we investigated the impact of different durations of sevoflurane on young adult mice cognitive performance, examined the expression of NR2B and the ratio of p-ERK1/2 to total ERK1/2 in the mice hippocampous following sevoflurane exposure. We also examined the activation of caspase3 and neuronal morphology in an attempt to understand the potential apoptosis induced by sevoflurane.

## Experimental Procedure

### Animals

This protocol was approved by the Shanghai Tongji University, School of Medicine, Animal Care and Use Committee. All experimental procedures were performed in accordance with the National Institutes of Health (NIH) guidelines for animal care (Guide for the Care and Use of Laboratory Animals, Department of Health and Human Services, NIH Publication No. 86–23, revised 1985).

Male C57BL/6 mice (4–5 weeks old) were obtained from Fudan University Animal Care Committee. All the mice were housed separately under standard laboratory conditions (12:12 light/dark cycle, 22°C, 60% humidity) with free access to tap water and food. The animals were allowed to adapt to laboratory environments for at least three weeks before experiments.

### Mice treatment

**Fig 1:**
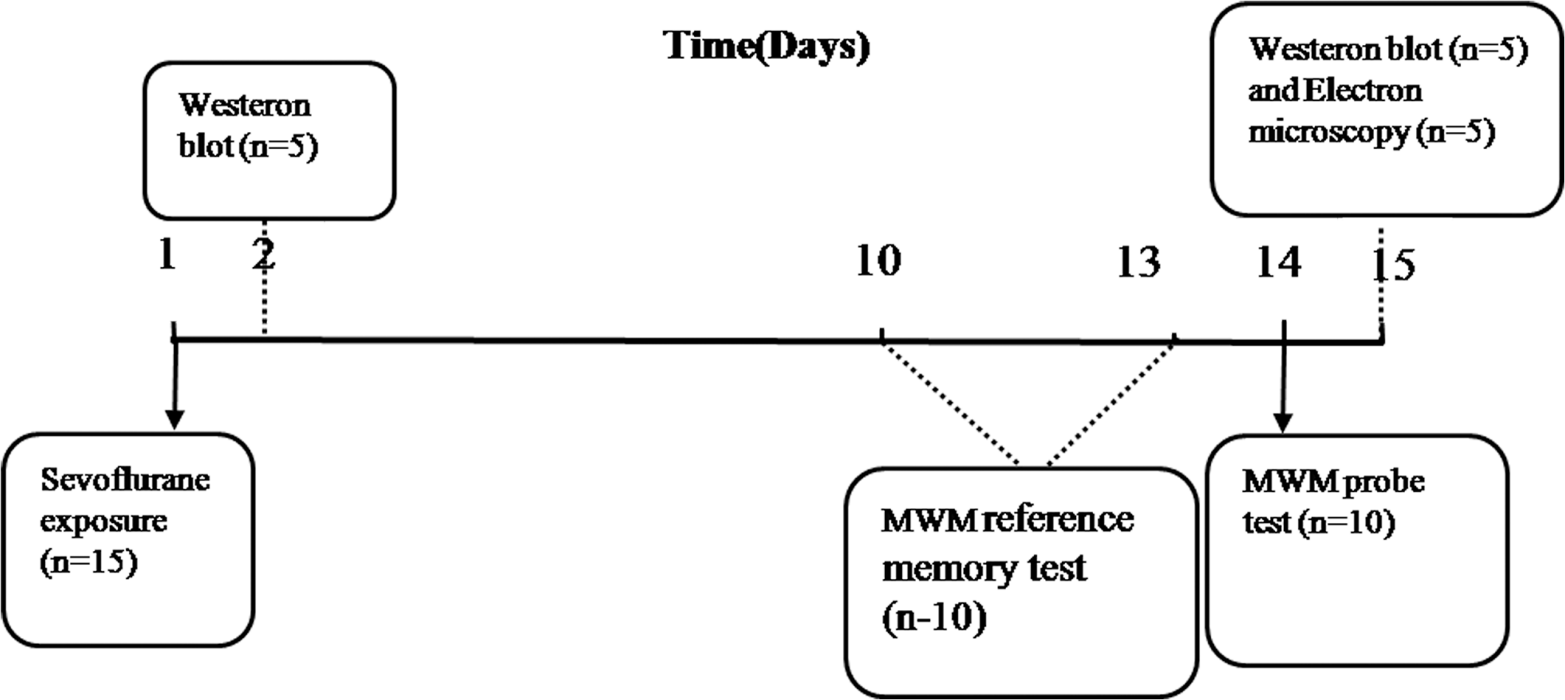
Schematic time-line of the experimental design.

Non-fasted mice (8 weeks old) were randomly divided into five groups (n = 15): control group (S0) and four anesthesia groups (S1, S2, S3 and S4). The anesthetized mice in S1, S2, S3 and S4 groups were placed in closed chambers and received sevoflurane plus air and oxygen (FiO_2_ = 0.5) at varying durations: 1, 2, 3 and 4h respectively. The concentration of sevoflurane was 2.5% in all anesthesia groups. The concentrations of sevoflurane and oxygen were monitored continuously (GE Datex-Ohmeda, Tewksbury, MA). Rectal temperature was maintained between 37 and 38°C by applying a warming blanket. To prevent hypoxia during sevoflurane exposure, we analyzed the blood gas levels in five additional mice at the end of 4 hours sevoflurane-exposure to avoid influence on cognitive and behavioural testing, which revealed normal oxygenation, adequate PCO2 values, and no acidosis (pO2: 125±10mmHg, pCO2: 38±5mmHg, pH: 7.38±0.01). After recovery, all experimental mice were returned to the raising cages. No mortality was observed during anesthesia and two weeks after anesthesia. Naïve mice S0 were kept in their home cage, in the same room as experimental animals for the duration of anesthetic exposure with no anesthesia. A schedule showing the time (days) of different experiments was provided (**Fig** 1).

### Morris Water Maze (MWM) test

The cognitive performance of the mice was assessed using the MWM test. MWM test is the most widely used technique in behavioral neuroscience for studying neural mechanisms of spatial learning and memory. The test was administered by an operator blinded to the group conditions. The MWM consisted of a round steel pool(122 cm in diameter and 60cm in height), which was filled with water to a level of 1 cm above the top of a platform (10 cm in diameter, 30 cm in depth). The pool was surrounded by a blue curtain with cues and was located in an isolated quiet room (20°C, 60% humidity). The water was maintained at 21°C and opacified with titanium dioxide.

The MWM test began at day 10 from the beginning of sevoflurane exposure and continued for five days (n = 10). The first four days (day 10–13) were the phase of place navigation test (reference memory test), which consisted of 16 training trials: four trials per day for four days with an inter-trial interval of 30–40 min. At the beginning of each trial, the animals were placed into water facing the wall, from different start positions (north, south, east, and west) and allowed 60 s to find the hidden platform and 15 s to stay on it. If the animal failed to locate the platform within 60 s, it was gently guided to the platform and allowed to stay on it for 15 s. A video tracking system was used to record the swimming activities of the animals. The escape latency (i.e., the time from dipping into water to staying on the platform) was recorded. On the fifth day (day 14), a spatial probe test was performed in which the platform was removed from the pool. The mouse was placed in the opposite quadrant and allowed to swim freely for 120s. The numbers of platform crossings were recorded.

Data were analyzed by using motion-detection software for the MWM (Shanghai Mobile Datum Information Technology Co, Ltd.).

### Antibodies and immunoblotting

Animals were killed by cervical dislocation, decapitated and their brains were rapidly removed at 24h post-anesthesia (n = 5), and at two weeks (after MWM test, n = 5). The control group was processed 24h after exposure. The bilateral hippocampi were dissected with 4°C saline and frozen in liquid nitrogen within 3 min of decapitation, and stored at -80°C. Antibodies to NR2B (ab28373, Abcam, USA), pERK1/2 (ab115617, Abcam, USA), ERK1/2(ab17942, Abcam, USA), Caspase3 (ab2302, Abcam, USA) and β-actin (Sigma-Aldrich, USA) were used. The total proteins were extracted and concentrations were measured by using bicinchoninic acid (BCA) protein determination assay (Pierce, Rockford, IL). Samples (25 μg protein) were resolved in 8% sodium dodecyl sulfate (SDS)-polyacrylamide gels and transferred to nitrocellulose membrane. The nitrocellulose membrane was then saturated in 5% skim milk solution for 1h and incubated overnight at 4°C with anti-NR2B (1:1000), anti-pERK1/2 (1:1000), anti-ERK1/2 (1:1000), and anti-Caspase3 (1:1000) in 5% skim milk solution. The membrane was rinsed 3 times (5 min each) in 0.05% Tween-Tris buffered solution (TTBS) buffer and incubated with alkaline phosphatase-conjugated secondary antibody (1:5000, Burlingame, CA) for 1 h at room temperature. The membrane was washed and processed by using the enhanced chemiluminescence method. Visualization and quantification of band density were performed with AlphaView Software3.4.

### Electron microscopy

Two weeks post-anesthesia, mice in each group (n = 5) were anesthetized with pentobarbital and perfused transcardially with 4% paraformaldehyde and 2% glutaraldehyde (Sigma-Aldrich, USA) in 0.01% phosphate buffer saline (PBS). The brain tissues were dissected and post-fixed in 2% glutaraldehyde at 4°C for 2 h. The brain was transversely sliced into 1-mm thick slabs. Blocks measuring 1×1×1 mm3 were severed from the hippocampus, post-fixed in 1% osmium tetroxide, dehydrated in graded alcohol, cleared in toluene, and embedded in araldite. The slabs were then sectioned into ultrathin slices, stained with uranyl acetate and lead citrate. The ultrastructure was observed and photographed by using a transmission electron microscopy (Philips CM 120, The Netherlands).

### Statistical analysis

Data were expressed as mean ± SD. Statistical comparisons between experimental and control groups were performed by one-way analysis of variance (ANOVA) with Least-significant difference post-hoc analysis, or repeated measures analysis of variance depending on outcome measure using statistics software SPSS (version 14.0; USA). A value of P < 0.05 was used for statistical significance.

## Results

### Sevoflurane induced changes of spatial cognition

**Fig 2:**
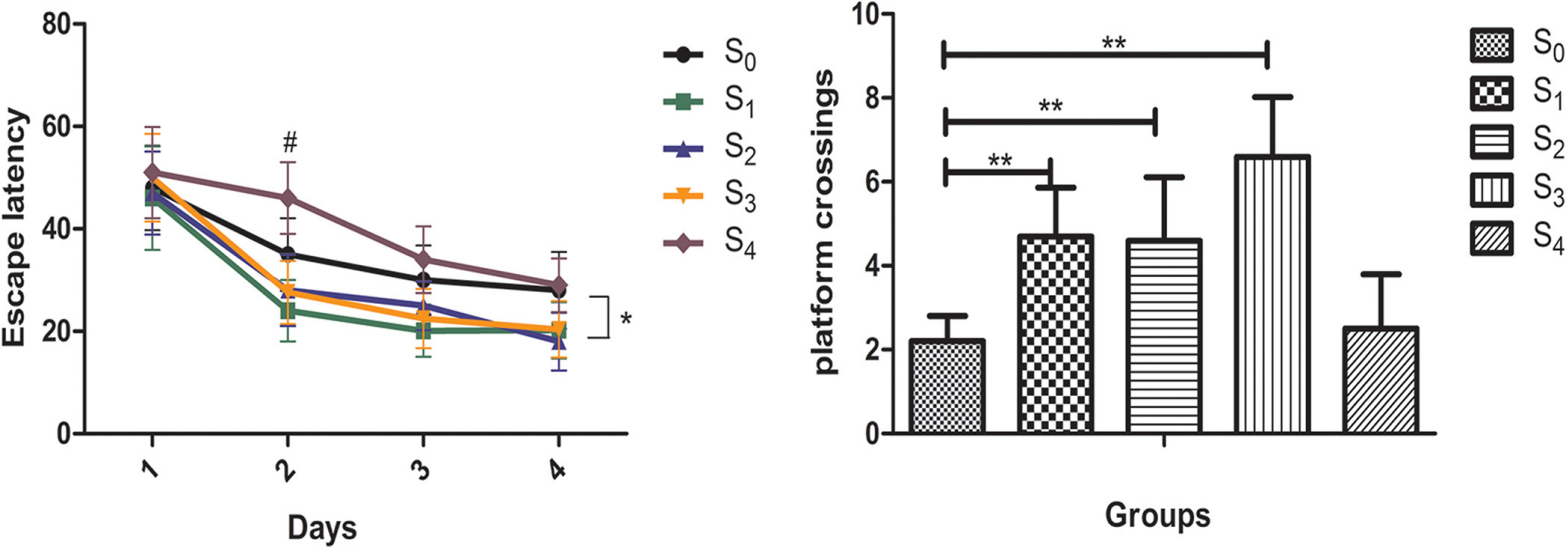
Sevoflurane anesthesia induced mice spatial cognitive changes. (A) The mice in S1, S2 and S3 groups exhibited significantly shorter escape latency than that in the control group. No significant difference was noted between S4 group and the control group except that on the second day of MWM test S4 had longer escape latency. (B) Platform crossings were increased in S1, S2 and S3 groups but not in S4 group as compared to control group (*: *P* < 0.05, **: *P* < 0.01; ^#^: p<0.05, S4 VS S0)

To evaluate the spatial memory effects of sevoflurane on the mice, Morris Water Maze(MWM) was performed 10 days post the sevoflurane exposure.(n = 10). In the place navigation test, the escape latency of each mouse was recorded. The mice in S1, S2 and S3 groups exhibited significantly shorter escape latency than that in the control group (1st day: F = 1.333, P = 0.062; 2nd day: F = 6.062, P = 0.031; 3rd day: F = 12.253, P = 0.032; 4th day: F = 3.011, P = 0.028). However, Mice in S4 showed longer escape latency than that in the control on the second day (**Fig 2A**). In spatial probe test we found that mice in S1, S2 and S3 groups crossed the platform significantly more than that in the control group(F = 10.841, P = 0.000). No difference was observed in platform crossings between S4 and control group(P = 0.065) (**Fig 2B**). No significant differences in swimming speed were noticed between anesthetic groups and control group (data not shown). Data indicated that treatment with 2.5% sevoflurane within three hours induced cognitive improvement.

### Sevoflurane induced upregulation of NR2B in the hippocampus

**Fig 3:**
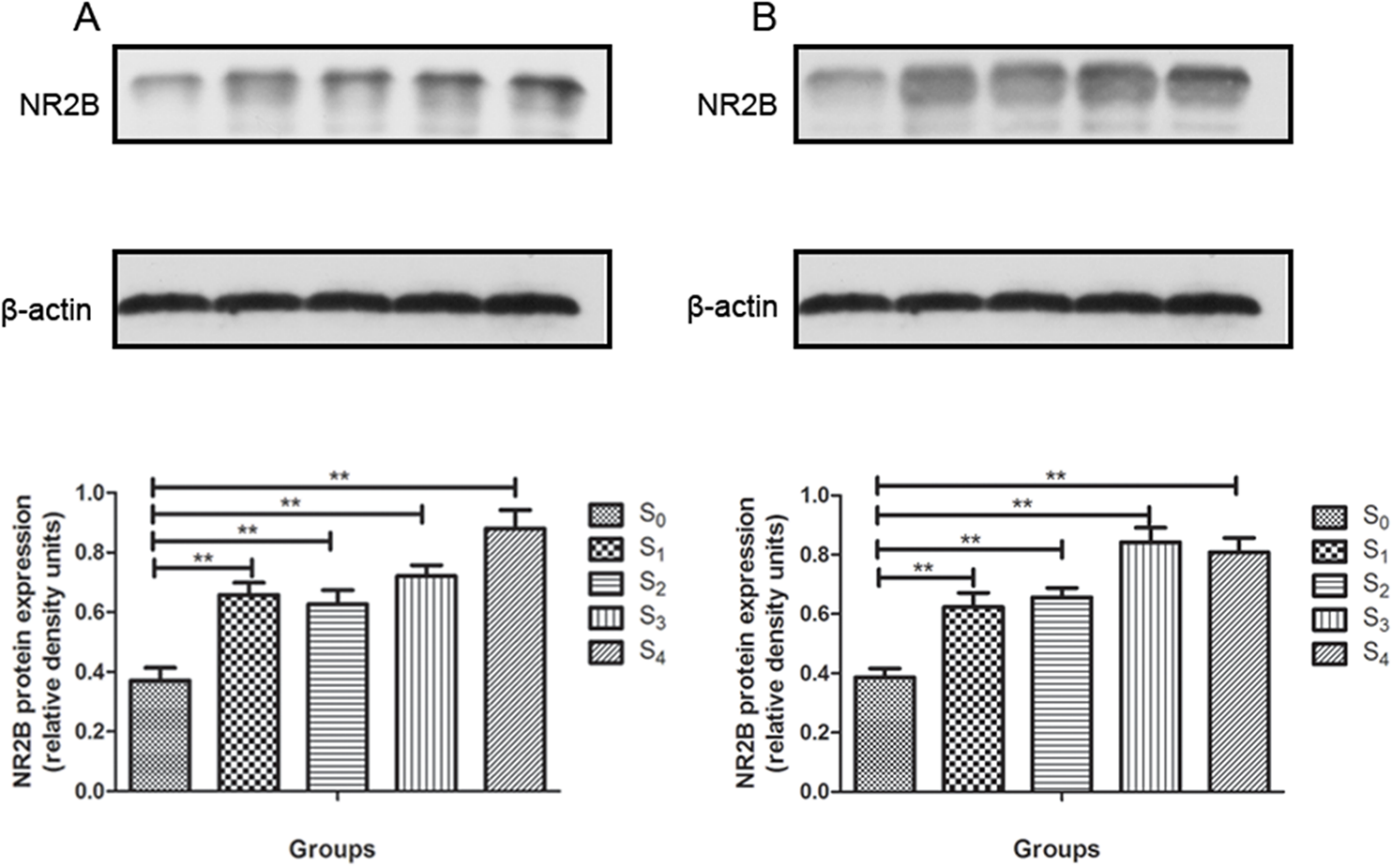
Effects of sevoflurane anesthesia on early and late protein expression of NR2B in the mice hippocampus. NR2B expression was increased in all sevoflurane-treated groups at 24 hours post- anesthesia (A) and 2 weeks post-anesthesia (B). Data are presented as mean ± SD. (*P < 0.05, **P < 0.01).

Hippocampal samples from different groups were homogenized and the expression of NR2B was determined. The level of NR2B expression was assessed at 24 h and two weeks post-sevoflurane treatment, when the animals completed the MWM behavioral analysis, in order to examine an early and late changes after anesthesia treatment. NR2B expression was increased in all anesthesia groups (S1 to S4) compared to the control group (S0) both at early(F = 77.048,P = 0.000) (**Fig 3A**) and late time (F = 93.511,P = 0.000) (**Fig 3B**). Therefore, sevoflurane exposure may induce NR2B expression increased for at least two weeks.

### Sevoflurane activated ERK1/2 in the hippocampus

**Fig 4:**
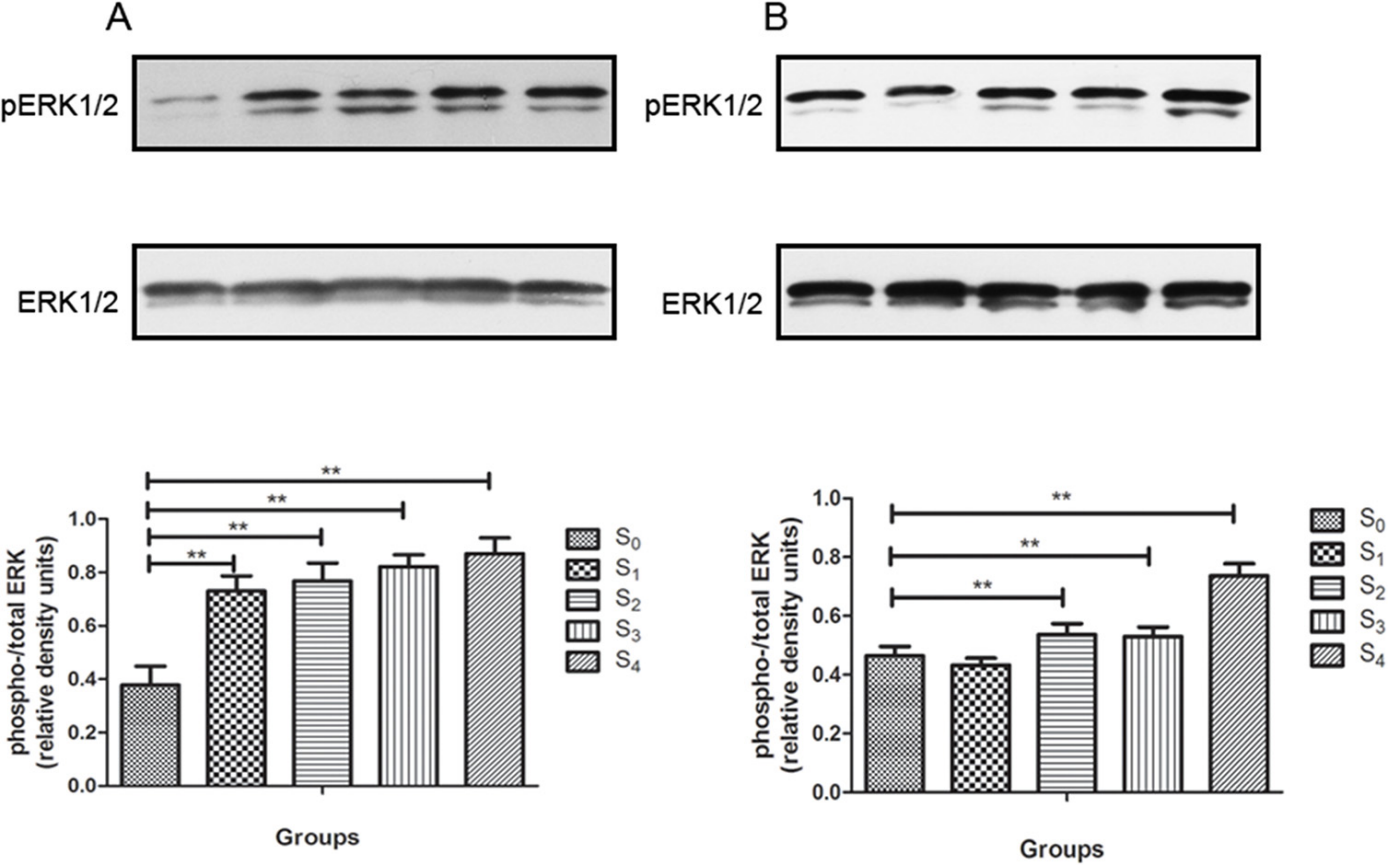
Effects of sevoflurane anesthesia on early and late ERK1/2 activation in the mouse hippocampus. (A) Activated ERK1/2 was increased in all sevoflurane-treated groups compared to control group at 24 hours post-anesthesia (*P* < 0.01). (B) Activated ERK1/2 was increased in S2, S3 and S4 groups at 2 weeks post-anesthesia. Data are presented as mean ± SD.(*P < 0.05, **P < 0.01).

The p-ERK1/2 and total ERK1/2 levels in the hippocampal tissues were determined at 24h and two weeks post-treatment, when the animals completed the MWM behavioral analysis. The ratio of p-ERK1/2 and total ERK1/2 determines change in activation of ERK1/2. The p-ERK1/2: total ERK ratio increased in all anesthesia groups (S1 to S4) as compared to control (S0) at 24 h after sevoflurane exposure (F = 51.907,P = 0.000) **(Fig 4A**). The p-ERK1/2: total ERK ratio also increased in S2 to S4 groups but not in S1 group as compared to control (S0) at two weeks later (F = 59.696,P = 0.000)(**Fig 4B**). Thus, sevoflurane activated ERK1/2 within 24 h after exposure up to four hours. The increased p-ERK1/2: total ERK1/2 ratio was still detected in the groups with two- to four-hour exposure.

### Sevoflurane activates caspase3 in the hippocampus

**Fig 5:**
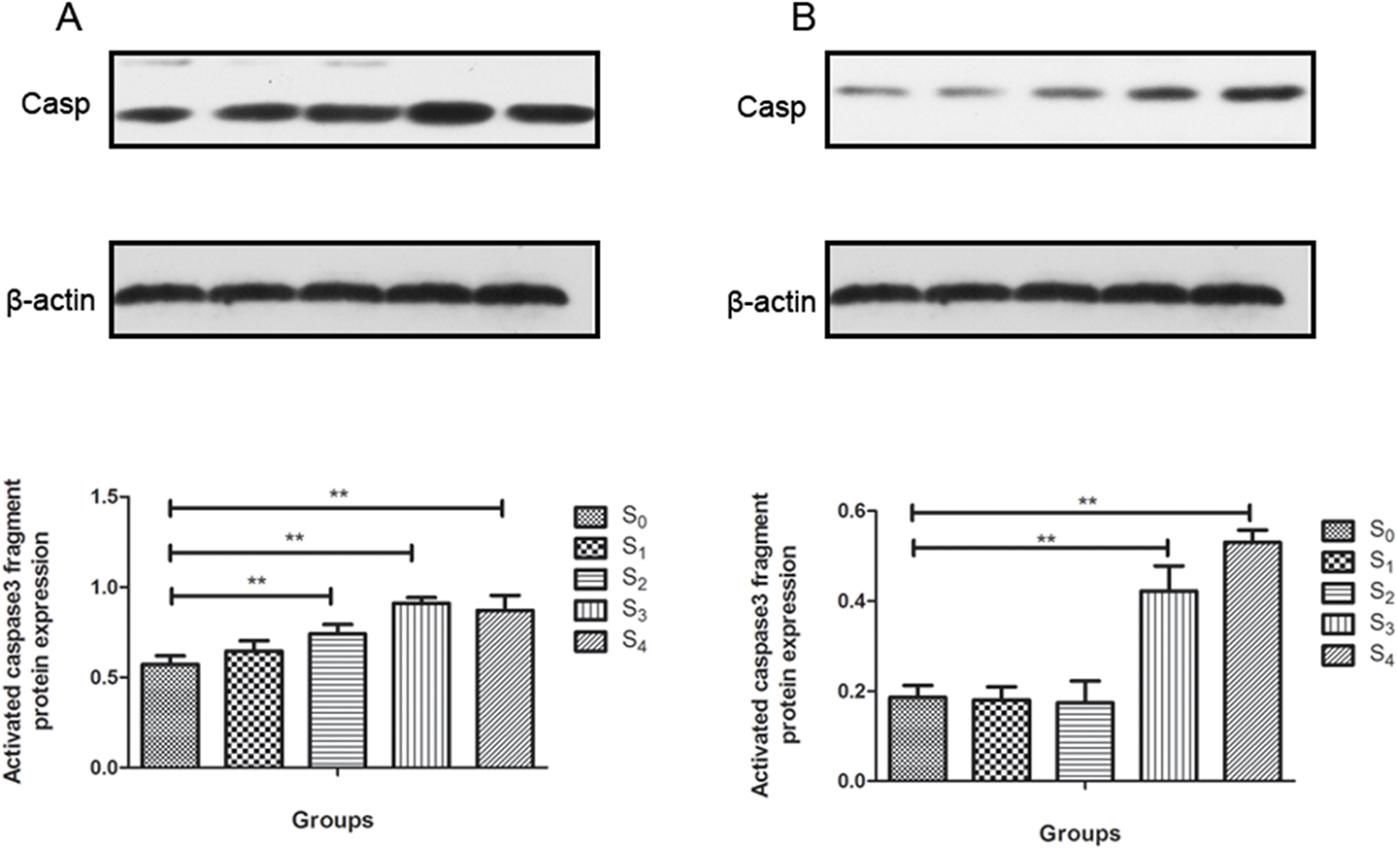
Effects of sevoflurane anesthesia on early and late caspase3 activation in the mouse hippocampus. (A) The activated caspase3 was increased in S2, S3 and S4 groups at 24 hours post-anesthesia compared to control group. (B) The activated caspase3 was increased in S3 and S4 groups at 2 weeks post- anesthesia compared to control group. Data are presented as mean ± SD. (*P < 0.05, **P < 0.01).

We quantified the 17kD fragment of activated caspase3 in the hippocampus in all groups. The quantification was performed twice: at 24 h and at two weeks after sevoflurane treatment. At 24 h post-anesthesia, caspase3 was elevated in S2, S3 and S4 groups but not in S1 group as compared to control group(F = 31.372,P = 0.000) (**Fig 5A**). Two weeks later, caspase3 was still elevated in S3 and S4 groups as compared to control group(F = 89.783, P = 0.000) (**Fig 5B**). Therefore, two- to four-hour sevoflurane exposure activated caspase3 at 24 h post anesthesia. However, only three- to four-hour sevoflurane exposure caused activation of caspase3 for at least two weeks post-anesthesia.

### Four-hour sevoflurane exposure induced apoptosis

**Fig 6:**
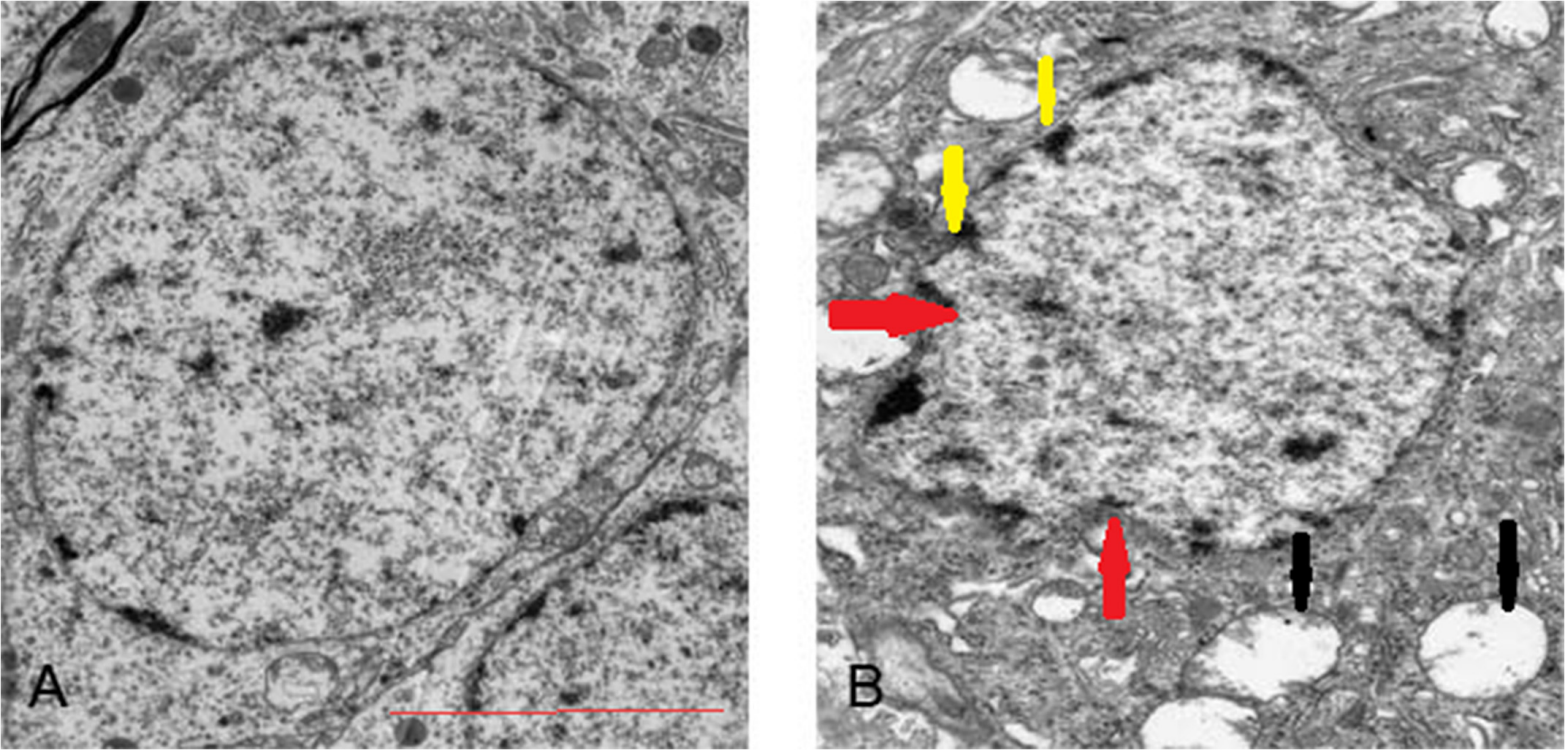
Transmission electron microscopic images. (A) normal brain cell in control (B) apoptotic cell in S4 brain. Ultrastructural changes include mitochondrial swelling, disintegrating mitochondrial cristae (black arrow), nucleus membrane shrinkage (red arrow) and chromatin margination (yellow arrow) by ultrahistological analysis. The scale bar represents 5μm.

The typical apoptotic changes were absent in the hippocampal neurons in control (**Fig 6A**), S1, S2 and S3 group (data not shown) at two weeks later. Normal cellular morphology including intact nuclear membrane, evenly distributed chromatin and clear ultrastructural findings were present. The typical apoptotic cells were observed in the hippocampus of the S4 group two weeks later. Ultrastructural changes including mitochondrial swelling, vanishing mitochondrial cristae, shrunken nucleus membrane and chromatin margination were seen in ultrahistological analysis (**Fig 6B**). Therefore, four-hour sevoflurane exposure induced apoptosis in the hippocampus.

## Discussion

General anesthesia affects learning and memory via impairment or improvement in cognitive function[1–5]. In this study, we found that 2.5% sevoflurane exposure within 3 hours can improve young adult mice spatial cognition two weeks later. Crosby et,al reported that 1.2% isoflurane-70% nitrous oxide for 2 hours improved maze performance 2 weeks later in adult mice. In addition, we also found that sevoflurane exposure increased the levels of hippocampal NMDA receptor subunit NR2B and the activation of ERK1/2. As we know, N-methyl-Daspartate (NMDA) receptors and downstream signaling pathways are very critical in learning and memory. A previous study reported that sevoflurane selectively reduces the expression of NMDA receptor synaptic subunit NR2A and increases the expression of extra-synaptic subunit NR2B in neonatal rats[18]. Therefore, together with the improvement of cognitive behavior after sevoflurane treatment, we presented NR2B and downstream signaling molecules playing a possible role for sevoflurane in improving spatial learning and memory in mouse.

It is well known that hippocampus plays a vital role in learning and memory and is a target for drugs modulating cognitive processes[20]. We evaluated the levels of NR2B subunit and ERK1/2 in mouse hippocampus as the expression of NR2B and ERK1/2 in hippocampus was closely related with hippocampus-dependent spatial learning and memory tasks[21]. In adult hippocampus and neocortex, NR2A and NR2B are the predominant NR2 subunits. The balance between synaptic subunit NR2A and extra-synaptic subunit NR2B determines the fate of neurons[22]. An increase of NR2B is associated with neuronal death or neurodegeneration. NR2A inhibition together with NR2B activation shifts the role of NMDA receptors from neuroprotection to excitotoxicity [23,24]. However, NR2B also appears to be a target for enhancement of memory and LTP. Activation of NR2B is considered a feasible strategy for the modification of synaptic plasticity and enhancement of learning and memory [25,26]. The overexpression of NR2B subunit in transgenic mice is associated with enhanced activation of NMDA receptors and improved learning and memory[27]. The induction of long-term potentiation (LTP) correlates with increased NR2B levels in the dentate gyrus of rats[28]. Our data indicated the beneficial role of increased NR2B levels in memory enhancement accompanied by improved spatial cognition.

In this study, the levels of p-ERK1/2 ratio were up-regulated by sevoflurane treatment. Although, upregulation of p-ERK1/2 is contrary to the reported inhibition of the phosphorylation of ERK1/2 by sevoflurane treatment[18]. However, considering that sevoflurane also increases NR2B expression, and that the NR2B subunit can both negatively and positively activate ERK signaling in the cortical neurons in rat depending on the developmental stages [29], it is plausible that upregulation of p-ERK1/2 and increased expression of NR2B co-exist after sevoflurane treatment. Increased p-ERK1/2 levels promote growth and survival of neurons[17]. p-ERK1/2 activates specific transcription factors, modulates expression levels and functions of key synaptic proteins [30,31]. This process affects many forms of synaptic plasticity, including long-term potentiation (LTP) and long-term depression (LTD), cellular models of learning and memory [32,33]. Therefore, the finding that sevoflurane induced the upregulation of p-ERK1/2 might also be related with improved spatial cognitive function.

As we know, learning and memory processes are very complicated, which can be affected by many factors. Neuronal death through apoptosis may cause the deficits in consolidating learning and memory. It is reported that anesthetic-induced cognitive impairment is related to neural apoptosis resulting from caspase3 activation in the CNS[34,35]. Caspase3, when activated by proteolytic cleavage, is one of the apoptotic effectors responsible for breakdown of cellular components. Activated caspase3 is widely used as a marker for apoptosis [36]. Zhang and colleagues demonstrated that sevoflurane inhalation activates caspase3 and enhances neuronal apoptosis in the developing brains of mice [37]. In this study, we also found that 3–4h treatment with 2.5% sevoflurane increased the activated caspase3 levels in hippocampus. The activation of caspase3 persisted for at least two weeks in group S3 and S4. Substantial apoptosis in group S4 was confirmed by EM. Given that the cognitive improvement of other sevoflurane exposure groups, the longest sevoflurane exposure mice in S4 performed similarly to control group, which may be the results of apoptosis induced by long-term sevoflurane treatment. We speculatd that the 4 hour sevoflurane treatment could induce neural toxicity in mice, which offset the facilitative effects on cognition induced by upregulation of hippocampal NR2B and p-ERK1/2. Thus, exceeding 4h exposure to 2.5% sevoflurane induced neuronal apoptosis and might eventually result in impairment of spatial cognition in mice.

## Conclusion

Our findings indicated that sevoflurane exposure with limited duration increases the levels of hippocampal NR2B and p-ERK1/2, potentially contributing to the improvement of spatial learning and memory. However, relatively long-term sevoflurane exposure induces neuronal apoptosis through activation of caspase3. We concluded that limited exposure to sevoflurane exerts a beneficial effect on cognitive performance. This might be due to the persistent effect on NR2B and ERK1/2 activation. However, prolonged exposure might lead to functional impairment and neurotoxicity.

## References

[1] Culley DJ, Baxter M, Yukhananov R, Crosby G. The memory effects of general anesthesia persist for weeks in young and aged rats. Anesth Analg. 2003; 96: 1004–1009. 12651650 10.1213/01.ANE.0000052712.67573.12

[2] Culley DJ, Baxter MG, Crosby CA, Yukhananov R, Crosby G. Impaired acquisition of spatial memory 2 weeks after isoflurane and isoflurane-nitrous oxide anesthesia in aged rats. Anesth Analg. 2004; 99: 1393–1397. 15502036 10.1213/01.ANE.0000135408.14319.CC

[3] Culley DJ, Baxter MG, Yukhananov R, Crosby G. Long-term impairment of acquisition of a spatial memory task following isoflurane-nitrous oxide anesthesia in rats. Anesthesiology. 2004; 100: 309–314. 14739805 10.1097/00000542-200402000-00020

[4] Komatsu H, Nogaya J, Anabuki D, Yokono S, Kinoshita H, Shirakawa Y et al Memory facilitation by posttraining exposure to halothane, enflurane, and isoflurane in ddN mice. Anesth Analg. 1993; 76: 609–612. 8452275 10.1213/00000539-199303000-00028

[5] Rammes G, Starker LK, Haseneder R, Berkmann J, Plack A, Zieglgänsberger W, et al Isoflurane anaesthesia reversibly improves cognitive function and long-term potentiation (LTP) via an up-regulation in NMDA receptor 2B subunit expression. Neuropharmacology. 2009; 56: 626–636 10.1016/j.neuropharm.2008.11.002 19059421

[6] Alkire MT, Gruver R, Miller J, McReynolds JR, Hahn EL, Cahill L. Neuroimaging analysis of an anesthetic gas that blocks human emotional memory. Proc Natl Acad Sci USA. 2008;105:1722–1727. 10.1073/pnas.0711651105 18227504 PMC2234211

[7] Sakai EM, Connolly LA, Klauck JA. Inhalation anesthesiology and volatile liquid anesthetics: focus on isoflurane, desflurane, and sevoflurane. Pharmacotherapy. 2005; 25: 1773–88 16305297 10.1592/phco.2005.25.12.1773

[8] Gibert S, Sabourdin N, Louvet N, Moutard ML, Piat V, Guye ML, et al Epileptogenic effect of sevoflurane: determination of the minimal alveolar concentration of sevoflurane associated with major epileptoid signs in Children. Anesthesiology. 2012; 117: 1253–1261. 10.1097/ALN.0b013e318273e272 23103557

[9] Edwards DA, Shah HP, Cao W, Gravenstein N, Seubert CN, Martynyuk AE. Bumetanide alleviates epileptogenic and neurotoxic effects of sevoflurane in neonatal rat brain. Anesthesiology. 2010; 112: 567–575. 10.1097/ALN.0b013e3181cf9138 20124973

[10] Satomoto M, Satoh Y, Terui K, Miyao H, Takishima K, Ito M, et al Neonatal exposure to sevoflurane induces abnormal social behaviors and deficits in fear conditioning in mice. Anesthesiology. 2009; 110: 628–637. 10.1097/ALN.0b013e3181974fa2 19212262

[11] Fang F, Xue Z, Cang J. Sevoflurane exposure in 7-day-old rats affects neurogenesis, neurodegeneration and neurocognitive function. Neurosci. 2012; 28: 499–508. 10.1007/s12264-012-1260-4 PMC5561912 22965743

[12] Feng X, Liu JJ, Zhou X, Song FH, Yang XY, Chen XS, et al Single sevoflurane exposure decreases neuronal nitric oxide synthase levels in the hippocampus of developing rats. Br J Anaesth. 2012; 109: 225–233 10.1093/bja/aes121 22535834 PMC3393078

[13] Haseneder R, Starker L, Berkmann J, Kellermann K, Jungwirth B, Blobner M, et al Sevoflurane Anesthesia Improves Cognitive Performance in Mice, but Does Not Influence In Vitro Long-Term Potentation in Hippocampus CA1 Stratum Radiatum. PLoS One. 2013; 8(5):e64732 10.1371/journal.pone.0064732 23724087 PMC3665835

[14] Alkire MT, Hudetz AG, Tononi G. Consciousness and anesthesia. Science. 2008; 322: 876–880. 10.1126/science.1149213 18988836 PMC2743249

[15] Solt K, Forman SA. Correlating the clinical actions and molecular mechanisms of general anesthetics. Curr Opin Anaesthesiol. 2007; 20: 300–306. 17620835 10.1097/ACO.0b013e32816678a5

[16] Léveillé F, El Gaamouch F, Gouix E, Lecocq M, Lobner D, Nicole O, et al Neuronal viability is controlled by a functional relation between synaptic and extrasynaptic NMDA receptors. FASEB J. 2008; 22: 4258–4271. 10.1096/fj.08-107268 18711223

[17] Ivanov A, Pellegrino C, Rama S, Dumalska I, Salyha Y, Ben-Ari Y, et al Opposing role of synaptic and extrasynaptic NMDA receptors in regulation of the extracellular signal-regulated kinases (ERK) activity in cultured rat hippocampal neurons. J Physiol. 2006; 572: 789–798. 16513670 10.1113/jphysiol.2006.105510 PMC1779993

[18] Wang WY, Jia LJ, Luo Y, Zhang HH, Cai F, Mao H, et al Location- and Subunit-Specific NMDA Receptors Determine the Developmental Sevoflurane Neurotoxicity Through ERK1/2 Signaling. Mol Neurobiol. 2014; 10.1007/S12035-014-9005-1 25421211

[19] Morgado-Bernal I. Learning and memory consolidation: linking molecular and behavioral data. Neuroscience. 2011; 176: 12–19. 10.1016/j.neuroscience.2010.12.056 21215299

[20] Jarrard LE. On the role of the hippocampus in learning and memory in the rat. Behav Neural Biol. 1993: 60: 9–26. 8216164 10.1016/0163-1047(93)90664-4

[21] Selcher JC, Atkins CM, Trzaskos JM, Paylor R, Sweatt JD. 1999 A necessity for MAP kinase activation in mammalian spatial learning. Learn. Mem. 6, 478–490. 10541468 10.1101/lm.6.5.478 PMC311312

[22] Hardingham GE, Bading H. Synaptic versus extrasynaptic NMDA receptor signalling: implications for neurodegenerative disorders. Nat Rev Neurosci. 2010; 11: 682–696. 10.1038/nrn2911 20842175 PMC2948541

[23] Liu Y, Wong TP, Aarts M, Rooyakkers A, Liu L, Lai TW, et al NMDA receptor subunits have differential roles in mediating excitotoxic neuronal death both in vitro and in vivo. Neurosci. 2007; 27: 2846–2857. 10.1523/JNEUROSCI.0116-07.2007 PMC6672582 17360906

[24] Zhou M, Baudry M. Developmental changes in NMDA neurotoxicity reflect developmental changes in subunit composition of NMDA receptors. Neurosci. 2006; 26: 2956–2963. 10.1523/JNEUROSCI.4299-05.2006 PMC6673978 16540573

[25] Bibb JA, Mayford MR, Tsien JZ, Alberini CM. Cognition enhancement strategies. J. Neurosci. 2010; 30: 14987–14992. 10.1523/JNEUROSCI.4419-10.2010 21068302 PMC3425350

[26] Collingridge GL, Volianskis A, Bannister N, France G, Hanna L, Mercier M, et al The NMDA receptor as a target for cognitive enhancement. Neuropharmacology. 2013; 64: 13–26. 10.1016/j.neuropharm.2012.06.051 22796429 PMC4696548

[27] Tang YP, Shimizu E, Dube GR, Rampon C, Kerchner G.A, Zhuo M, et al Genetic enhancement of learning and memory in mice. Nature. 1999; 401: 63–69. 10485705 10.1038/43432

[28] Williams JM, Mason-Parker SE, Abraham WC, Tate WP. Biphasic changes in the levels of N-methyl-D-aspartate receptor-2 subunits correlate with the induction and persistence of long-term potentiation. Brain Res Mol Brain Res. 1998; 60: 21–27. 9748484 10.1016/s0169-328x(98)00154-5

[29] Sava A, Formaggio E, Carignani C, Andreetta F, Bettini E, Griffante C. NMDA-induced ERK signalling is mediated by NR2B subunit in rat cortical neurons and switches from positive to negative depending on stage of development. Neuropharmacology. 2012; 62: 925–932. 10.1016/j.neuropharm.2011.09.025 22001284

[30] Sgambato V, Vanhoutte P, Pagès C, Rogard M, Hipskind R, Besson MJ, et al In vivo expression and regulation of Elk-1, a target of the extracellular-regulated kinase signaling pathway, in the adult rat brain. J Neurosci. 1998; 18: 214–226. 9412502 10.1523/JNEUROSCI.18-01-00214.1998 PMC6793414

[31] Mao L, Tang Q, Samdani S, Liu Z, Wang JQ. Regulation of MAPK/ERK phosphorylation via ionotropic glutamate receptors in cultured rat striatal neurons. Eur J Neurosci. 2004; 19: 1207–1216. 15016079 10.1111/j.1460-9568.2004.03223.x

[32] Sweatt JD. Mitogen-activated protein kinases in synaptic plasticity and memory. Curr Opin Neurobiol. 2004; 14: 311–317. 15194111 10.1016/j.conb.2004.04.001

[33] Thomas GM, Huganir RL. MAPK cascade signalling and synaptic plasticity. Nat Rev Neurosci. 2004; 5: 173–183. 14976517 10.1038/nrn1346

[34] Xie Z, Dong Y, Maeda U, Alfille P, Culley DJ, Crosby G, et al The common inhalation anesthetic isoflurane induces apoptosis and increases amyloid beta protein levels. Anesthesiology. 2006; 104: 988–994. 16645451 10.1097/00000542-200605000-00015

[35] Xie Z, Culley DJ, Dong Y, Zhang G, Zhang B, Moir RD, et al The common inhalation anesthetic isoflurane induces caspase activation and increases amyloid beta-protein level in vivo. Ann Neurol. 2008; 64: 618–627. 10.1002/ana.21548 19006075 PMC2612087

[36] Young C, Roth KA, Klocke BJ, West T, Holtzman DM, Labruyere J, et al Role of caspase3 in ethanol-induced developmental neurodegeneration. Neurobiol Dis. 2005; 20: 608–614. 15927478 10.1016/j.nbd.2005.04.014

[37] Zhang X, Xue Z, Sun A. Subclinical concentration of sevoflurane potentiates neuronal apoptosis in the developing C57BL/6 mouse brain. Neurosci Lett. 2008; 447: 109–114. 10.1016/j.neulet.2008.09.083 18852026

